# Gray matter structural networks related to ^18^F-THK5351 retention in cognitively normal older adults and Alzheimer's disease patients

**DOI:** 10.1016/j.ensci.2021.100309

**Published:** 2021-01-07

**Authors:** Yoko Shigemoto, Daichi Sone, Kyoji Okita, Norihide Maikusa, Tensho Yamao, Yukio Kimura, Fumio Suzuki, Hiroyuki Fujii, Koichi Kato, Noriko Sato, Hiroshi Matsuda

**Affiliations:** a Department of Radiology, National Center of Neurology and Psychiatry, 4-1-1, Ogawa-Higashi, Kodaira, Tokyo 187-8551, Japan; b Integrative Brain Imaging Center, National Center of Neurology and Psychiatry, 4-1-1, Ogawa-Higashi, Kodaira, Tokyo 187-8551, Japan; c Department of Clinical and Experimental Epilepsy, UCL Institute of Neurology, University College London, Queen Square, London WC1N 3BG, United Kingdom; d Department of Drug Dependence Research, National Institute of Mental Health, National Center of Neurology and Psychiatry, 4-1-1, Ogawa-Higashi, Kodaira, Tokyo 187-8551, Japan; e Cyclotron and Drug Discovery Research Center, Southern TOHOKU Research Institute for Neuroscience, Koriyama 963-8052, Japan

**Keywords:** Alzheimer's disease, Graph theory, Gray matter, Magnetic resonance imaging, Single-subject

## Abstract

**Objective:**

This study aimed to examine the alterations in gray matter networks related to tau retention in Alzheimer's disease (AD) patients and cognitively normal (CN) older individuals.

**Methods:**

Eighteen amyloid-positive AD patients and 30 age- and sex-matched amyloid-negative CN controls were enrolled. All underwent 3D T1-weighted MRI, amyloid positron-emission tomography imaging (PET) with ^11^C-Pittsburgh Compound B (PiB), and tau PET with ^18^F-THK5351. The structural networks extracted from the T1-weighted MRI data based on cortical similarities within single subjects were analyzed. Based on graph theoretical approach, global and local network properties across the whole brain were computed. Group comparisons of global and local network properties were evaluated between the groups. Then, we correlated the global and local network measures with total cerebral ^18^F-THK5351 retention.

**Results:**

AD patients moved toward more randomized global network compared to controls and regional differences were observed in the default mode network (DMN) area. No significant correlations existed between global network properties and tau retention. On a local level, AD and controls showed opposite relationships between network properties and tau retention mainly in the DMN areas; CN controls showed positive correlations, whereas AD showed negative correlations.

**Conclusion:**

We found opposite relationships between local network properties and tau retention between amyloid-positive AD patients and amyloid-negative controls. Our findings suggest that the presence of amyloid and induced exacerbated tau retention alter the relationship of local network properties and tau retention.

## Highlights

• Correlation of structural network properties and tau retention.
• Positive correlations between local network properties and tau retention in healthy elderly.
• Negative correlations between local network properties and tau retention in AD.

## 1 Introduction

Alzheimer's disease (AD) is characterized by the presence of extracellular amyloid-β (Aβ) plaques and intracellular neurofibrillary tangles composed of hyperphosphorylated tau [1]. There is evidence for disconnection in AD, and these proteins are associated with local synaptic disruptions [2]. Graph theoretical analyses have been conducted by many studies to study brain networks in AD [3] using several neuroimaging modalities such as functional magnetic resonance imaging (fMRI), electroencephalography, and magnetoencephalography [[4], [5], [6], [7], [8], [9]]. More recently, structural network analysis using diffusion tensor imaging (DTI) or T1-weighted imaging has attracted attention [[10], [11], [12], [13], [14], [15], [16], [17], [18]]. Previous studies on structural networks using T1-weighted images are based on cortical thickness or volume across individuals and restricted to group-level analyses [11,13,14,17]. However, a recently proposed method has enabled interindividual-level analysis based on cortical similarities in gray matter (GM) morphology within single subjects [15,16,[19], [20], [21], [22]]. In this method, graphs were defined with nodes representing small cortical regions and edges representing connecting regions which have high statistical similarity. This method has been applied in AD, people at risk for schizophrenia, posttraumatic stress disorder, and infants with intrauterine growth restriction [[20], [21], [22]].

The recent advent of tau positron emission tomography (PET) has permitted the in vivo assessment of tau pathology, in addition to that of Aβ. Several studies have used resting-state fMRI to examine the interactive effects of these aggregated proteins on network changes, but their results were inconsistent [[23], [24], [25], [26]]. For instance, one study found positive associations between functional connectivity and tau-PET uptake both in amyloid-negative healthy elderly and amyloid-positive AD patients [23]. Another study reported positive associations between functional connectivity and CSF tau in amyloid-negative individuals with subjective memory complaints and controls [24]. However, Sepulcre et al. reported a negative association between tau and functional connectivity, whereas a positive association between amyloid and functional connectivity in the aging brain [25]. In terms of structural connectivity, to our knowledge, only two studies have investigated a possible interaction effect of Aβ and tau. One recent DTI study linked decreased connectivity of the hippocampal cingulum bundle to ^18^F-AV1451 retention in the posterior cingulate cortex in preclinical AD individuals but not in amyloid-negative CN individuals [27].

We recently reported that diffusion connectometry measured by DTI exhibited a completely opposite response to cerebral ^18^F-THK5351 retention: increased connectivity of tracks in amyloid-negative CN older individuals but decreased connectivity in amyloid-positive mild cognitive impairment (MCI) individuals and patients with early AD [28]. Diffusion connectometry tracks only the consecutive fiber which shows significant positive or negative correlations with study variables after measuring the connectivity between adjacent voxels within a white matter fiber comparing the density of diffusion spins [29]. This method is superior to conventional DTI analysis in that it overcame the problem of partial volume effects or crossing fibers [30]. According to the amyloid cascade hypothesis, the deposition of cortical amyloid is the causative agent of AD pathology and induce the tau deposition, cell loss, vascular damage, and lead to dementia [31]. Autopsy studies have shown no cerebral amyloid deposition in CN older individuals, but broad distribution of amyloid throughout the cerebral cortex in AD [32]. Whereas neuropathological studies for tau have revealed localized deposition in the medial temporal lobes (MTL) even in CN older individuals. In AD, it is suggested that the presence of cortical amyloid may exacerbate tau deposition in the MTL and spread into lateral temporal lobes beyond the collateral sulcus [33]. Our previous findings suggested that localized MTL tau may induce compensatory response (positive correlations between tau and connectivity) in CN older individuals, however the presence of cortical amyloid exacerbate the tau deposition and consequently induced breakdown of compensatory response (negative correlations between tau and connectivity) in AD [28].

Motivated by our previous findings using diffusion connectomery [28], we examined whether the correlation between tau and network properties derived from GM show the opposite relationship between the two groups. We hypothesized (1) that tau retention is significantly different between AD and controls and that (2) network properties are significantly different between AD and controls on a global and local level. Moreover, we hypothesized that (3) there is a significant correlation between network properties and tau retention which shows opposite relationship between AD and controls on a global and local level.

## 2 Materials and methods

### 2.1 Participants

We recruited 18 patients with AD and 30 age- and sex-matched CN older individuals from Brain Mapping by Integrated Neurotechnologies for Disease Studies (Brain/MINDS). All participants underwent 3 T structural MRI, amyloid PET imaging with ^11^C-Pittsburgh Compound B (PiB), tau PET imaging with ^18^F-THK5351, and neuropsychological testing.

Inclusion criteria for AD was based on the clinical criteria for probable AD outlined in the National Institute on Aging–Alzheimer's Association guidelines [34] and the presence of abnormal cortical amyloid retention as detected by visual assessment of ^11^C-PiB PET scans. Exclusion criteria was having contra-indication for MRI scans.

Inclusion criteria for controls was visually negative ^11^C-PiB PET scans, a global Clinical Dementia Rating (CDR) score of 0, Mini-Mental State Examination (MMSE) > 26, and performance within education-adjusted norms for the Wechsler Memory Scale-Revised Logical Memory II (WMSR LM-II). Exclusion criteria was having a neurological or psychiatric disorder, having medications that could interfere with cognition, and contra-indication for MRI scans.

All procedures performed in the studies were in accordance with the ethical standards of the institutional and/or national research committee and with the 1964 Helsinki declaration. All participants provided written informed consent to participate in the study, which was approved by the institutional review board at the National Center of Neurology and Psychiatry Hospital (A2014-146).

### 2.2 Image acquisition

All participants underwent structural MRI scans on a Siemens Verio 3 T scanner (Siemens, Erlangen, Germany). Three-dimensional (3D) sagittal T1-weighted magnetization-prepared rapid acquisition with gradient echo images were acquired in the same manner as in previous studies (repetition time/echo time, 1.900/2.52 ms; 1.0-mm effective slice thickness with no gap; 300 slices; matrix, 256 × 256; field of view, 25 cm × 25 cm; acquisition time; 4 min 18 s) [28,35].

^11^C-PiB and ^18^F-THK5351 were prepared and PET data were acquired as previously reported [28,35] using a Siemens/Biograph TruePoint 16 scanner (3D acquisition mode; 81 image planes; axial field of view, 16.2 cm; transaxial resolution, 4.2 mm; axial resolution, 4.7 mm; slice interval, 2 mm). ^11^C-PiB PET scans were acquired as dynamic scans using LIST mode 50–70 min after a bolus injection of 555 ± 185 MBq of ^11^C-PiB. ^18^F-THK5351 scans were acquired as dynamic scans using LIST mode 40–60 min after a bolus injection of 185 ± 37 MBq of ^18^F-THK5351. Low-dose computed tomography (CT) scans for attenuation correction were performed. PET/CT data were reconstructed using an iterative 3D ordered subset expectation maximization reconstruction algorithm.

### 2.3 MRI and PET data preprocessing

All 3D T1-weighted images were segmented into GM, white matter, and cerebrospinal fluid images using Statistical Parametric Mapping Software version 12 (SPM; Functional Imaging Laboratory, University College London, London, UK) implemented in MATLAB 7.12. The segmented GM images were normalized using the Diffeomorphic Anatomic Registration Through Exponentiated Lie (DARTEL) algebra algorithm and modulated using non-linear deformation [36]. After partial volume correction with PETPVE12 toolbox [37], each ^18^F-THK5351 PET image was normalized and coregistered to its individual T1-weighted image and normalized using DARTEL. Then, each PET image was warped using the deformation fields derived from DARTEL registration of the coregistered T1-weighted image to the reference template. MRI and PET data were preprocessed in the same manner as previously described [28,35].

After spatial normalization, we calculated the partial volume-corrected standardized uptake value ratios (SUVRs) of the ^18^F-THK5351 images using the individual's positive mean uptake value of cerebellar GM as a reference [28, 35]. To investigate the correlation of network measures and GM volume with ^18^F-THK5351 retentions, we calculated the SUVR in the total cerebrum using the Automated Anatomical Labeling atlas implemented in Wake Forest University PickAtlas, version 2.4 [38], as previously reported [28].

### 2.4 Single-subject GM networks

We extracted single-subject GM networks based on intracortical similarity using a previously described fully automated method (https://github.com/bettytijms/Single_Subject_Grey_Matter_Networks; version 20,150,902) [19]. Nodes are defined as small regions of interest in the brain (3 × 3 × 3 voxel cubes, corresponding to 6 mm × 6 mm × 6 mm). Each node was rotated by a θ angle with multiples of 45° and reflected over all axes to identify the maximal similarity value with the target node. Connectivity was defined by high statistical similarities quantified with Pearson's correlations across the GM density values of corresponding voxels between any two nodes. The similarity between all nodes within a single-subject scan was computed with the correlation coefficient. Next, to construct unweighted and undirected graphs, the similarity matrices were binarized with a threshold that ensured a 5% chance of spurious correlations for all single-subjects (corresponding to a *p* < .05 corrected for multiple comparisons by a false discovery rate technique using an empirical null distribution) [39]. Then, the network was binarized based on the determined threshold: a correlation greater than the threshold was indicated as 1 (i.e. 2 nodes were connected) and a correlation lower than the threshold was indicated as 0 (i.e. 2 nodes were not connected). In this study, connectivity is used to show there is high statistical similarity between any two nodes, which exist in the absence of axonal connectivity.

#### 2.4.1 Local network properties

To enable to compare our findings with those reported in previous studies, we decided to investigate the following basic network properties: betweenness centrality (BC), clustering coefficient (C), characteristic path length (L), and degree (k). All these network properties were calculated at local level.

##### 2.4.1.1 Betweenness centrality (BC)

The betweenness centrality *BC*_*i*_ denotes the proportion of shortest paths *s*_*j, m*_ between nodes *v*_*j*_ and *v*_*m*_ that run through a node *v*_*i*_ in the total network *G* [40].

$$BC_{i}=\sum_{i\neq j\neq m\in G}\frac{s_{j,m}\left(i\right)}{s_{j,m}}$$

##### 2.4.1.2 Clustering coefficient (C)

Clustering coefficient *c*_*i*_ of a node *v*_*i*_ is denoted as the number of edges *k*_*j*_ between the neighbors divided by the total number of possible edges *k*_*g*_*i*__ in subgraph *g*_*i*_ [41].

$$c_{i}=\frac{\sum_{j,k\in g_{i}}k_{j}}{k_{g_{i}}\left(k_{g_{i}}-1\right)/2}$$

##### 2.4.1.3 Characteristic path length (L)

The shortest path length *L*_*i*_ is defined as the minimum number of edges between pairs of node *v*_*i*_ and *v*_*j*_ [41].

$$L_{i}=\frac{\sum_{j=1,i\neq j}^{N}L_{i,j}}{N}$$

##### 2.4.1.4 Degree (k)

The degree *k* denotes the number of connections each node *v* has.

#### 2.4.2 Global network properties

The small world property *(δ)* of a binary graph is defined by global network of clustering *C*_*network*_ and path length *L*_*network*_ comparing each of these parameters of randomized network [42].

The clustering coefficient of the global network *C*_*network*_ is the average clustering coefficient *c*_*i*_ over all *N* nodes.

$$C_{\mathrm{network}}=\frac{\sum_{i=1}^{N}c_{i}}{N}$$

The shortest path length of the global network *L*_*network*_ is the average shortest path length *L*_*i*_ over all *N* nodes.

$$L_{\mathrm{network}}=\frac{\sum_{i=1}^{N}L_{i}}{N}$$

We calculated five randomized networks (μ = 5) for each individual's binarized similarity matrix with an identical size and degree distribution of *C*_*network*_ and *L*_*network*_ [43]. An average $\bar{C}$_*random*_ and $\bar{L}$_*random*_ were computed.

$$\;{\bar{C}}_{\mathrm{network}}=\frac{1}{\mu \sum_{i=1}^{\mu }C_{{\mathrm{random}}_{i}}}\;{\bar{L}}_{\mathrm{network}}=\frac{1}{\mu \sum_{i=1}^{\mu }C_{{\mathrm{random}}_{i}}}$$

The division of *C*_*network*_ by $\bar{C}$_*random*_ is denoted by γ, and the division of *L*_*network*_ by $\bar{L}$_*random*_ is denoted by λ [41,42].

$$\gamma =\frac{C_{\mathrm{network}}}{{\bar{C}}_{\mathrm{random}}}\;\lambda =\frac{L_{\mathrm{network}}}{{\bar{L}}_{\mathrm{random}}}$$

The small world property *δ* is denoted as the division of γ and λ [42]. $\sigma =\frac{\gamma }{\lambda }$

In CN subjects, brain network is considered to maintain efficient a “small world” network between regular and random network balancing both integration and segregation [44]. With these definitions, “small world” network is required to have *δ* > 1, γ > 1, and λ ~ 1 [42].

**Fig. 1:**
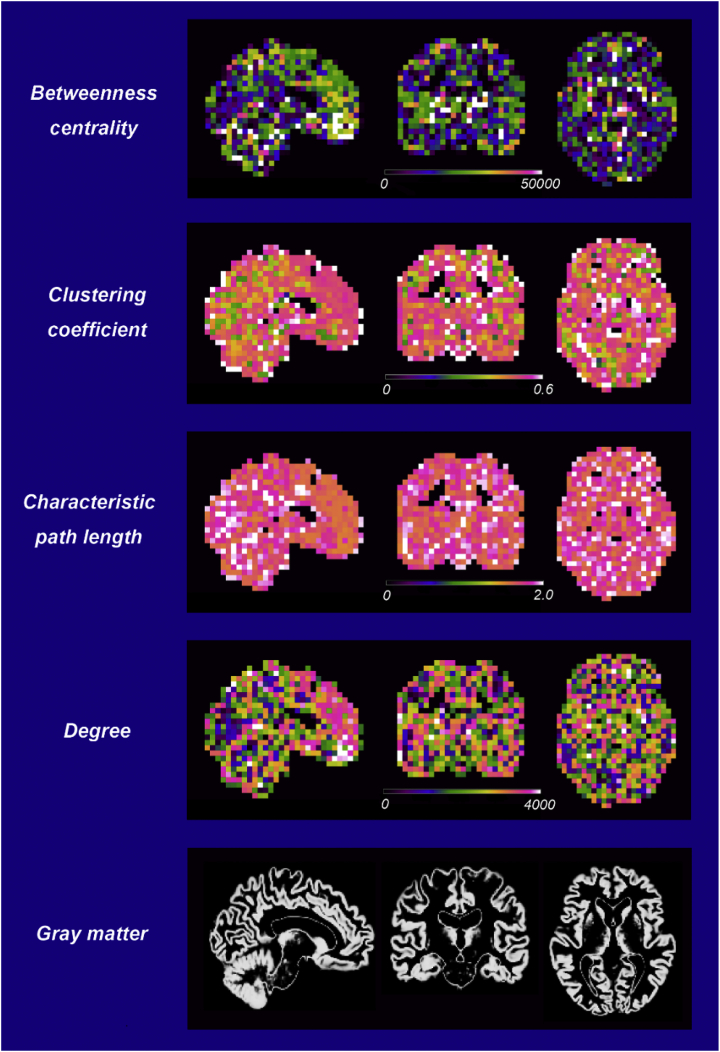
Examples of four single-subject local network graphs (betweenness centrality, clustering coefficient, characteristic path length, and degree) and resliced gray matter images. Each network metric was superimposed on the resliced gray matter images of Montreal Neurological Institute standard space. The color bar represents the absolute values.

To compare each participant's local network measures at the cubic level, we superimposed the corresponding images on the resliced GM of standard Montreal Neurological Institute space. An example of the local network measures of a CN older individual is shown in Fig. 1. Since this analysis is based on a large cube with 6 mm × 6 mm × 6 mm, the local network measures of gray matter may partly overlap on anatomical location of the white matter and ventricles.

Finally, each participant's local network measure images were smoothed using a 10-mm FWHM Gaussian kernel in the same manner as in the previous study on single-subject structural networks [45]. This value (10 mm) was determined by nearly doubling the resolution of one side of a cube (6 mm).

### 2.5 Statistical analysis

#### 2.5.1 Demographics

The statistical analyses for demographics were performed using Statistical Package for Social Science software (SPSS version 25.0; Japan, Tokyo). Demographic variables were compared by an unpaired *t*-test for continuous variables and Pearson's χ^2^ test for categorical variables. A *p* value<.05 was deemed significant.

#### 2.5.2 The group differences of ^18^F-THK5351 retention

^18^F-THK5351 SUVR between the groups was compared by an unpaired *t*-test and *p* < .05 was deemed significant.

Group comparisons of ^18^F-THK5351 SUVR images were evaluated using a two-sample *t*-test analysis in SPM12 with age and sex as nuisance covariates. Statistically, a height threshold of *p* < .05 (family-wise error [FWE] corrected), and an extent threshold of 50 voxels were considered significant.

#### 2.5.3 The group differences of global network properties

The group comparisons of the global network properties (γ, λ and δ) were compared via analysis of covariance (ANCOVA) with age and sex as nuisance covariates and *p* < .05 was deemed significant.

#### 2.5.4 The group differences of local network properties

To assess differences of local network properties between the groups, we tested the smoothed local network images (betweenness centrality, clustering coefficient, characteristic path length, and degree) using a two-sample *t*-test analysis in SPM12 with age and sex as nuisance covariates. Statistically, a height threshold of *p* < .05 (FWE corrected), and an extent threshold of 50 voxels were considered significant.

#### 2.5.5 Correlations between global network properties and ^18^F-THK5351 retention

The correlation between the global network properties and ^18^F-THK5351 retention was assessed with a partial correlation analysis controlling for age and sex in each group using SPSS. A *p* value<.05 was deemed significant.

#### 2.5.6 Correlations between local network properties/GM volume and tau retention

We applied a multiple regression design and SPM12 in each group to investigate the correlations between local network properties and ^18^F-THK5351 retention/regional GM volume. Participants' ^18^F-THK5351 SUVR was considered the main covariate and age and sex were considered the nuisance covariates. Statistically, a height threshold of *p* < .05 (FWE corrected), and an extent threshold of 50 voxels were considered significant. In addition, to consider the subtle local network changes, we also reported all clusters thresholded by a height of *p* < .001 (uncorrected).

## 3 Results

### 3.1 Demographics

Participants' demographics are presented in Table 1. The AD group was 69.5 ± 8.7 years old (mean ± standard deviation), their global CDR ranged from 0.5 to 1.0, their average MMSE score was 22.1 ± 4.7, and their average WMSR LM-II score was 2.8 ± 3.8. The controls were 68.1 ± 6.5 years old, had an average MMSE score of 29.1 ± 1.1, and a WMSR LM-II score of 12.8 ± 3.1. No significant differences in mean age or sex were observed between the groups.

**Table 1:** Participants' demographic characteristics.

	AD	CN	*p*-value
Number of participants	18	30	
Sex (female/male)	12/6	20/10	1.00^a^
Age (years)	69.5 ± 8.7	68.1 ± 6.5	0.52^b^
CDR	0.5–1.0	0	
MMSE score	22.1 ± 4.7	29.1 ± 1.1	<0.001^b^
WMSR LM-II score	2.8 ± 3.8	12.8 ± 3.1	<0.001^b^

AD, Alzheimer's disease; CDR, Clinical Dementia Rating; CN, cognitively normal; MMSE, Mini-Mental State Examination; WMSR LM-II, Wechsler Memory Scale-Revised Logical Memory II.

Data are means ± standard deviation.

a Pearson's χ^2^ test.

b unpaired *t*-test.

### 3.2 The group differences of ^18^F-THK5351 retention

The average ^18^F-THK5351 SUVR in the total cerebrum was 1.14 ± 0.17 for the CN older individuals and 1.49 ± 0.43 for the patients with AD. The AD group showed significantly higher ^18^F-THK5351 SUVR compared to the CN group (*p* = .001).

**Fig. 2:**
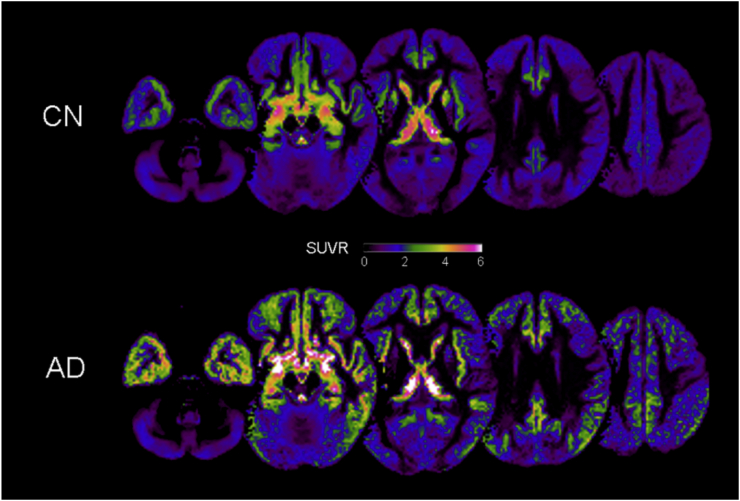
The average standardized uptake value images of ^18^F-THK5351 in the CN group and the AD group. The CN group exhibited localized ^18^F-THK5351 retention mainly in the basal ganglia, thalami, and medial temporal lobes, whereas the AD group showed more widely distributed and elevated tracer retention. The most striking differences of ^18^F-THK5351 retention between the groups were the medial temporal lobes. AD, Alzheimer's disease; CN, cognitively normal.

Fig. 2 shows the distribution of ^18^F-THK5351 retention. In the CN older individuals, localized ^18^F-THK5351 retention was mainly seen in the bilateral basal ganglia, thalami, and medial temporal lobes but also slightly extended into the inferior temporal lobes, insula, posterior cingulate gyri/precuneus, and basal frontal lobes. In contrast, the patients with AD showed more widely distributed and elevated tracer retention in these areas and in the inferior parietal lobes. The most striking differences in ^18^F-THK5351 retention between the two groups were in the bilateral MTL. Statistically, the AD group showed significantly increased ^18^F-THK5351 retention in the left parahippocampal gyrus (cluster size, 68 voxels; *Z*-score, 4.54; Talairach coordinate [x, y, z], [−22, −3, −18]).

### 3.3 The group differences of global network properties

**Fig. 3:**
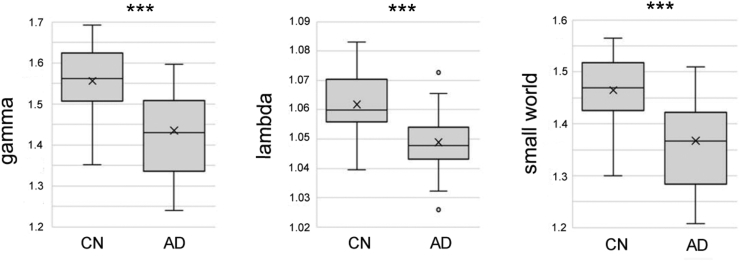
Distributions of global network properties in the CN older individuals and the patients with AD. AD, Alzheimer's disease; CN, cognitively normal. ***p < .001. The *p* values are corrected by ANCOVA.

The distributions of global network properties in CN older individuals and patients with AD are shown in Fig. 3. The average normalized clustering coefficients (γ), normalized characteristic path lengths (λ), and small-world values (δ) for CN older individuals and patients with AD were 1.556 ± 0.083 vs. 1.435 ± 0.104, 1.062 ± 0.115 vs. 1.049 ± 0.011, and 1.465 ± 0.064 vs. 1.367 ± 0.086, respectively. The AD group showed significantly lower global network properties than the CN group (all *p* < .001, ANCOVA).

### 3.4 The group differences of local network properties

**Fig. 4:**
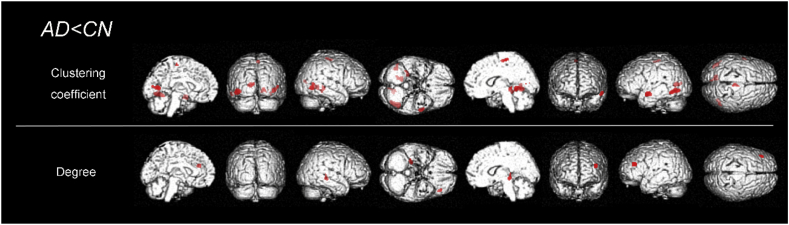
The distribution of reduced clustering coefficient and degree in the AD group compared to the CN group. The clustering coefficient was significantly reduced in the fusiform gyrus, posterior cingulate, middle temporal gyrus and lingual gyrus. The parahippocampal gyrus and middle frontal gyrus were reduced in degree. AD, Alzheimer's disease; CN, cognitively normal.

Table 2 and Fig. 4 show specific anatomical regions where the clustering coefficient was significantly reduced in the default mode network (DMN) area (posterior cingulate), temporal and occipital areas in the AD group compared to the CN group. The degree also showed reduced DMN area (parahippocampal gyrus) and frontal area.

**Table 2:** The group differences of local network measures between the AD group and the CN group.

	Cluster size	*t* value	x, y, z	Regions with peaks	
*AD* *<* *CN*					
Betweenness centrality	*Not significant*				
Clustering coefficient	234	7.23	−42, −57, −6	L	Fusiform gyrus
	157	6.78	−12, −66, 11	L	Posterior cingulate^a^
	93	6.70	−53, −3, −12	L	Middle temporal gyrus
	130	6.37	42, −60, −2	R	Middle temporal gyrus
	133	6.12	16, −47, −3	R	Lingual gyrus
Characteristic path length	*Not significant*				
Degree	78	6.75	26, −29, −4	R	Parahippocampal gyrus^a^
	68	6.52	−38, 36, 22	L	Middle frontal gyrus
CN < AD	*Not significant*				

The results are based on a height threshold of *p* < .05 (FWE-corrected), and an extent threshold of 50 voxels. The coordinates are those of the Talairach atlas. L, left; R, right.

AD, Alzheimer's disease; CN, cognitively normal.

a The areas of default mode network.

### 3.5 Correlations between global network properties and ^18^F-THK5351 retention

There were no significant correlations between the global network properties and ^18^F-THK5351 retention in the CN group (γ: *p* = .796; λ: *p* = .357; δ: *p* = .574) and the AD group (γ: *p* = .895; λ: *p* = .482; δ: *p* = .921).

### 3.6 Correlations between local network properties and ^18^F-THK5351 retention

**Fig. 5:**
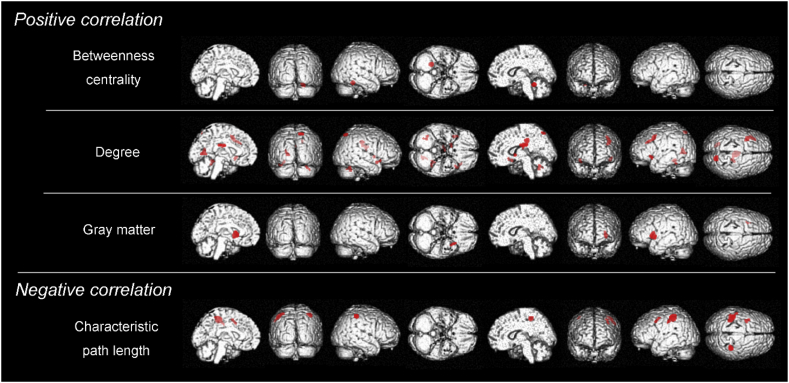
In the cognitively normal older individuals, positive correlations were evident between some of the local network measures/gray matter volume and ^18^F-THK5351 retention. Positive correlation between ^18^F-THK5351 retention and betweenness centrality was observed in the cerebellum. Degree also showed positive correlations in the cingulate gyrus, superior parietal lobule, middle/inferior frontal gyrus, posterior cingulate, fusiform gyrus, lentiform nucleus, and cerebellum. GM volume showed positive correlations in the claustrum. Characteristic path length showed negative correlations in the middle frontal gyrus, postcentral gyrus, and cerebellum.

**Fig. 6:**
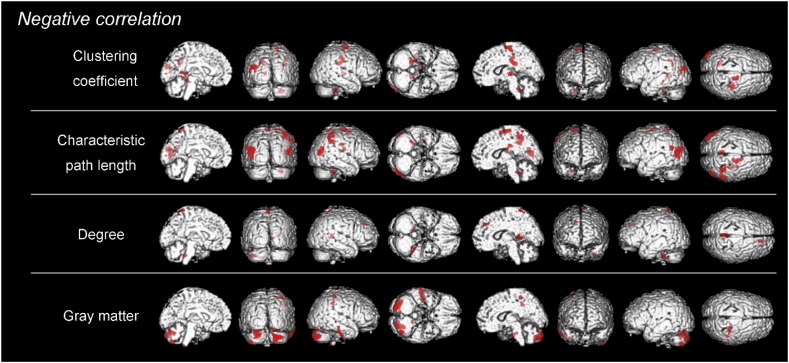
In the patients with Alzheimer's disease, negative correlations were observed between most of the local network measures/gray matter volume and ^18^F-THK5351 retention. Negative correlations between ^18^F-THK5351 retention and clustering coefficient in the in the superior occipital gyrus, medial frontal gyrus, posterior cingulate, precuneus, and thalamus. Characteristic path length showed negative correlations in the in the middle temporal gyrus, inferior parietal lobule, middle frontal gyrus, insula, posterior cingulate, superior temporal gyrus, precuneus and cerebellum. Degree also showed negative correlations in the superior fontal gyrus, posterior cingulate, and postcentral gyrus. GM volume showed negative correlations in the postcentral gyrus, inferior temporal gyrus, inferior parietal lobule, and cerebellum.

At the conservative level of *p* < .05 (FWE corrected), there were no significant correlations between the local network properties/GM volumes and ^18^F-THK5351 retention. At the exploratory level of *p* < .001 (uncorrected) showed positive correlations between ^18^F-THK5351 retention and some of the local network properties (i.e., betweenness centrality and degree)/GM volume in the CN group (Table 3 and Fig. 5), whereas negative correlations between ^18^F-THK5351 retention and most of the local network properties (i.e., clustering coefficient, characteristic path length, and degree) in the CN group (Table 4 and Fig. 6)

**Table 3:** Correlations between local network measures/gray matter volumes and ^18^F-THK5351 retention in the cognitively normal older individuals.

	Cluster size	*t* value	x, y, z	Regions with peaks	
*Positive correlations*					
Betweenness centrality	124	4.51	24, −45, −15	R	Anterior lobe
Clustering coefficient	*Not significant*				
Characteristic path length	*Not significant*				
Degree	280	6.05	6, −20, 27	R	Cingulate gyrus
	112	5.81	14, −65, 57	R	Superior parietal lobule
	186	4.83	−32, 12, 42	L	Middle frontal gyrus
	63	4.81	−40, 15, −7	L	Inferior frontal gyrus
	142	4.68	−18, −64, 9	L	Posterior cingulate^a^
	60	4.36	16, 4, 0	R	Lentiform nucleus
	55	4.24	36, 11, −12	R	Inferior frontal gyrus
	77	4.20	−28, −41, −11	L	Fusiform gyrus
	65	4.10	38, −56, −26	R	Anterior lobe
Gray matter volume	333	4.28	−26, 16, 3	L	Claustrum
*Negative correlations*					
Betweenness centrality	*Not significant*				
Clustering coefficient	*Not significant*				
Characteristic path length	146	4.63	24, −45, −15	R	Anterior lobe
	57	4.49	−34, 10, 38	L	Middle frontal gyrus
	301	4.36	−40, −27, 49	L	Postcentral gyrus
Degree	*Not significant*				
Gray matter volume	*Not significant*				

The results are based on a height threshold of *p* < .001 (uncorrected) and an extent threshold of 50 voxels. The coordinates are those of the Talairach atlas. L, left; R, right.

a The areas of default mode network.

**Table 4:** Correlations between local network measures/gray matter volume and ^18^F-THK5351 retention in the patients with Alzheimer's disease.

	Cluster size	*t* value	x, y, z	Cerebral region	
*Positive correlations*					
Betweenness centrality	*Not significant*				
Clustering coefficient	*Not significant*				
Characteristic path length	*Not significant*				
Degree	*Not significant*				
Gray matter volume	*Not significant*				
*Negative correlations*					
Betweenness centrality	*Not significant*				
Clustering coefficient	128	8.85	−38, −84, 24	L	Superior occipital gyrus
	273	6.26	32, −22, 32	R	Frontal lobe, sub-gyral
	158	5.69	14, −9, 63	R	Medial frontal gyrus
	67	5.19	14, −19, 3	R	Thalamus
	96	5.01	−12, −48, 10	L	Posterior cingulate^a^
	82	4.92	−20, −55, 34	L	Precuneus^a^
	79	4.74	26, −40, −38	R	Cerebellar tonsil
Characteristic path length	459	6.97	−42, −75, 13	L	Middle temporal gyrus
	395	6.19	36, −46, 48	R	Inferior parietal lobule^a^
	184	6.03	18, −9, 61	R	Middle frontal gyrus
	126	5.91	32, −11, 19	R	Insula
	233	5.40	−18, −64, 9	L	Posterior cingulate^a^
	54	4.55	−53, −59, 25	L	Superior temporal gyrus
	52	4.54	26, −38, −27	R	Anterior lobe
	50	4.50	−4, −47, 63	L	Precuneus^a^
Degree	52	6.82	14, 41, 33	R	Superior frontal gyrus
	93	5.68	12, −44, 6	R	Posterior cingulate^a^
	50	5.60	−2, −45, 67	L	Postcentral gyrus
	58	5.06	−32, −38, −30	L	Anterior lobe
Gray matter volume	874	5.66	−26, −67, −20	L	Posterior lobe
	1213	5.54	24, −83, −26	R	Posterior lobe
	175	5.23	26, −32, 53	R	Postcentral gyrus
	246	4.94	55, −28, −25	R	Inferior temporal gyrus
	71	4.51	48, −37, 39	R	Inferior parietal lobule^a^

The results are based on a height threshold of *p* < .001 (uncorrected) and an extent threshold of 50 voxels. The coordinates are those of the Talairach atlas. L, left; R, right.

a The areas of default mode network.

In the CN group, positive correlation between ^18^F-THK5351 retention and betweenness centrality was observed in the cerebellum. Degree also showed positive correlations in the DMN area (posterior cingulate), frontal and parietal areas. Characteristic path length was observed to have no positive correlations but did have negative correlations in the frontal and parietal areas. Clustering coefficient had no positive or negative correlations. Additionally, positive correlations were found between ^18^F-THK5351 retention and GM volume in the claustrum.

In contrast, the AD group showed negative correlations between ^18^F-THK5351 retention and clustering coefficient in some of the DMN areas (posterior cingulate, precuneus), frontal and occipital areas. Characteristic path length showed negative correlations in the DMN areas (inferior parietal lobule, posterior cingulate, precuneus) and fronto-temporal areas. Degree also showed negative correlations in the DMN area (posterior cingulate), frontal and parietal areas. Betweenness centrality had no positive or negative correlations. Additionally, negative correlations were found between GM volume and ^18^F-THK5351 retention in the DMN area (inferior parietal lobule), parietal and temporal areas.

## 4 Discussion

In this study, we investigated the correlations between ^18^F-THK5351 retention and network properties in the CN and AD groups. Our analysis revealed positive correlations between local network measures and ^18^F-THK5351 retention in DMN area in the CN group, however negative correlations in these areas in the AD group. These opposite relationship between ^18^F-THK5351 retention and local network properties in AD compared to controls may induced by the presence of cortical amyloid and exacerbated tau retention.

### 4.1 The group differences of ^18^F-THK5351 retention

The CN older individuals showed elevated ^18^F-THK5351 retention compared to other cortical areas including sensory and motor areas where tau pathologically does not accumulate until the end stage of AD. This was present mainly in the medial temporal lobes but also slightly extended into the inferior temporal lobes, insula, posterior cingulate gyri/precuneus, and basal frontal lobes in Braak stage III–IV [1]. A recent large-cohort tau PET study [46] revealed elevated tracer retentions in Braak stage III–IV areas in the absence of Aβ and suggested the possibility of primary age-related tauopathy [47]. Our results might also be related to primary age-related tauopathy. However, we cannot rule out the influence of off-target binding of ^18^F-THK5351 to monoamine oxidase B (MAO-B) [[48], [49], [50]], whose level increases throughout the brain with human aging [51]. The patients with AD showed more widely elevated ^18^F-THK5351 retention consistent with Braak stage V–VI.

The group differences of ^18^F-THK5351 retention were visually evident in the MTL. Statistically, the AD group showed significantly increased ^18^F-THK5351 retention in the left parahippocampal gyrus compared to controls. Our finding was consistent with pathological staging of tau in that the AD patients show most severe tau depositions in the entorhinal and transentorhinal regions which are the anterior part of parahippocampal gyrus and the first area tau accumulates [1].

### 4.2 The group differences of global/local network properties

On a global level, both CN and AD groups showed a “small world” network which is defined by δ > 1, γ > 1, and λ ~ 1. However, the AD group showed significantly lower γ, λ, and δ compared to the CN group, indicating AD is moving toward random network which is in line with previous functional network studies [[5], [6], [7]] and GM structural network study in AD [15].

On a local level, the AD group revealed reduced local network properties in the specific areas which are known to be affected in the disease. The areas that showed reduced local network properties in AD included posterior cingulate, fusiform gyrus and lingual gyrus in clustering coefficient and parahippocampal gyrus in degree. These areas were previously reported to be disrupted in functional network [6,7] and group-based structural network studies [11,17] and in single-subject network study [15]. The posterior cingulate and parahippocampal gyrus are the part of DMN areas, which are considered the first areas to be affected in AD and to be involved in episodic memory and executive function [26].

### 4.3 Correlations between global/local network properties and ^18^F-THK5351 retention

On a global level, there were no significant correlations between network properties and ^18^F-THK5351 retention both in the CN and the AD group.

On a local level, although there were no significant correlations between network properties and ^18^F-THK5351 retention at the conservative level of *p* < .05 (FWE corrected), using a more liberal threshold of *p* < .001 (uncorrected) showed positive correlations in the CN group but negative correlations in the AD group. These findings are consistent with our previous diffusion connectometry results, which showed the completely opposite response to ^18^F-THK5351 retention; positive correlations between ^18^F-THK5351 retention and connectivity in the CN group, whereas negative correlations in the MCI/AD group [28].

As discussed in the previous study, we speculate that the positive correlations between local network properties and ^18^F-THK5351 retention in the amyloid-negative CN older individuals were caused by a compensatory response that aims to maintain normal cognition, namely tau alone acts in a protective manner. However, the compensatory mechanisms are considered to occur in response to Aβ toxicity or an amyloid-induced inflammatory response in preclinical or early AD [7,52,53]. The present findings may indicate that tau-induced compensatory responses also present even in NFT^+^/Aβ^−^ aged brains.

On the other hand, the negative correlations between local network properties and ^18^F-THK5351 retention observed in the amyloid-positive AD patients were possibly caused by the breakdown of compensatory response which was induced by the appearance of cortical amyloid and exacerbated tau deposition. We speculate that tau alone acts in a protective manner for cognition in the CN group showing positive correlations between network properties and tau, however the coexistence of amyloid and tau no longer have protective effects showing negative correlations between network properties and tau.

Although the responses of the two groups to network changes were opposite, the areas with network changes were similar. We found network changes in DMN areas [54]: the posterior cingulate in the CN controls and the posterior cingulate, inferior parietal lobule, and precuneus in the AD patients. Several fMRI studies have shown that tau pathology is related to the DMN [[24], [25], [26]]. Even in individuals with a subjective memory complaint, tau-related functional network changes have been detected in the DMN [24]. The same study showed that coexistence of hypoconnectivity in the DMN and hyperconnectivity in the medial temporal region acted as a compensatory mechanism to maintain normal cognition. Although the present study did not include individuals with a subjective memory complaint in the CN older group, the findings suggest that, even in amyloid-negative CN older individuals, tau-related compensatory effects may exist in DMN areas.

The areas with ^18^F-THK5351-related GM volume changes were different from the areas with network changes, except for some overlap in the patients with AD. We found tau-related positive GM volume changes in the claustrum but in none of the DMN areas in the CN older individuals. These findings suggest that structural network analysis derived from GM cortical similarities does not directly reflect cortical atrophy.

This study has several limitations. First, ^18^F-THK5351 reflects MAO-B-related astrogliosis in addition to tau pathology. Human blocking studies with the MAO-B inhibitor selegiline identified significantly decreased uptake even in the cortex, as well as in the thalamus [55]. Also, ^18^F-THK5351 is considered a biomarker of astrogliosis rather than tau pathology, so our findings possibly reflect a nonspecific neuroinflammation-related structural response, in addition to tau pathology. Second, the sample size was small for both the CN older individuals and the patients with AD. Third, this is a cross-sectional study, so longitudinal studies that investigate network changes over time are needed to prove the robustness of this method.

## 5 Conclusions

We investigated the correlations between ^18^F-THK5351 retention and network measures in the CN and the AD groups. The amyloid-negative CN group revealed positive correlations between local network measures and ^18^F-THK5351 retention in DMN area, however the amyloid-positive AD group showed negative correlations in these areas. These opposite relationship between ^18^F-THK5351 retention and local network measures observed in AD compared to controls may be induced by the presence of cortical amyloid and exacerbated tau retention. Our findings suggest that the presence of amyloid alters the relationship of local network measures and ^18^F-THK5351 retention. Single-subject GM network analysis may contribute to better understanding of the correlation of tau and network measures in AD.

## Grant support

This study was supported by the following funding sources: the Brain Mapping by Integrated Neurotechnologies for Disease Studies (Brain/MINDS) project (grant no. 18dm0207017h0005), funded by the 10.13039/100009619Japan Agency for Medical Research and Development (AMED), and an Intramural Research Grant (27-9) for Neurological and Psychiatric Disorders from the 10.13039/501100009438National Center of Neurology and Psychiatry (Japan).

## Declaration of Competing Interest

None.
